# Linagliptin in Combination With Metformin Ameliorates Diabetic Osteoporosis Through Modulating BMP-2 and Sclerostin in the High-Fat Diet Fed C57BL/6 Mice

**DOI:** 10.3389/fendo.2022.944323

**Published:** 2022-07-19

**Authors:** Nikita Nirwan, Divya Vohora

**Affiliations:** Neurobehavioral Pharmacology Laboratory, Department of Pharmacology, School of Pharmaceutical Education and Research, Jamia Hamdard, New Delhi, India

**Keywords:** DPP-4 inhibitors, linagliptin, metformin, diabetes, high fat diet (HFD), osteoporosis, bone architecture

## Abstract

**Background:**

Diabetic osteoporosis is a poorly managed serious skeletal complication, characterized by high fracture risk, increased bone resorption, reduced bone formation, and disrupted bone architecture. There is a need to investigate drugs that can improve bone health along with managing glycemic control. DPP-4 inhibitors and metformin have proven benefits in improving bone health. Here, we investigated the effects of linagliptin, a DPP inhibitor, and metformin alone and in combination to treat diabetic osteoporosis in high-fat-fed mice.

**Methods:**

C57BL/6 mice were kept on the high-fat diet (HFD) for 22 weeks to induce diabetic osteoporosis. Linagliptin (10mg/Kg), metformin (150mg/Kg), and their combination were orally administered to the diabetic mice from the 18^th^-22^nd^ week. Femur and tibial bone microarchitecture together with bone mineral density (BMD) were evaluated using µCT and histopathological changes were assessed. Further, bone turnover biomarkers namely bone morphogenetic protein-2 (BMP-2), sclerostin, tartrate-resistant acid phosphatase (TRAP), osteocalcin, alkaline phosphatase (ALP), calcium, and pro-inflammatory cytokines were assessed. Additionally, metabolic parameters including body weight, fasting blood glucose (FBG), glucose & insulin tolerance, lipids profile, and leptin were measured.

**Results:**

HFD feeding resulted in impaired bone microarchitecture, reduced BMD, distorted bone histology, and altered bone turnover biomarkers as indicated by the significant reduction in bone ALP, BMP-2, osteocalcin, and an increase in sclerostin, TRAP, and serum calcium. Interestingly, treatment with linagliptin and its combination with metformin significantly reverted the impaired bone architecture, BMD, and positively modulated bone turnover biomarkers, while metformin alone did not exhibit any significant improvement. Further, HFD induced diabetes and metabolic abnormalities (including an increase in body weight, FBG, impaired glucose and insulin tolerance, leptin, triglycerides, cholesterol), and pro-inflammatory cytokines (TNF-alpha and IL-1β) were successfully reversed by treatment with linagliptin, metformin, and their combination.

**Conclusion:**

Linagliptin and its combination with metformin successfully ameliorated diabetic osteoporosis in HFD-fed mice possibly through modulation of BMP-2 and sclerostin. The study provides the first evidence for the possible use of linagliptin and metformin combination for managing diabetic osteoporosis.

## Introduction

Diabetic osteoporosis is a relatively newer complication associated with type 2 diabetes mellitus which is characterized by high fracture risk, increase bone resorption, impaired bone formation, and disrupted bone architecture (1–4). Every year, more than 9 million fractures occur due to osteoporosis (5). According to a cohort study on Health, Aging, and Body Composition, diabetic adults experience about 64% of higher fracture risk as compared to non-diabetic adults with similar bone mineral density (BMD) (6).

Some of the plausible mechanisms behind diabetes mellitus associated bone deficits include hyperglycemia, increased production of advanced glycation end products (AGE), macrophage colony-stimulating factor, tumor necrosis factor (TNF), receptor activator of nuclear factor-B ligand (RANKL), and reduced expression of runt-related transcription factor (Runx)-2, osteocalcin, and osteopontin (7, 8). Hyperglycemia also modulates some important bone turnover biomarkers such as bone morphogenetic protein-2 (BMP-2), which promotes osteoblast differentiation, and sclerostin, which retard bone formation (9–11). Previous *in-vitro* studies indicate that hyperglycemia reduces BMP-2 levels in bone mesenchymal stem cells and increases sclerostin levels in murine cell lines (12, 13). The clinical report also confirms that circulating sclerostin level is high in type 2 diabetes patients (14). Hence, the use of therapeutic agents which could target BMP-2 and sclerostin might prove beneficial in improving diabetes-linked osteoporosis.

Anti-diabetic drugs may have a positive, negative, or neutral impact on bone health. Anti-diabetic drugs such as thiazolidinedione (TZD) and sodium-glucose transporter-2 (SGLT-2) inhibitors have a deleterious influence on bone health as signified by accelerating adipogenesis and increased fracture risk (15). Therefore, there is a need to investigate drugs that can improve bone health along with managing glycemic control in diabetic patients with poor bone health.

Preclinical studies indicate that incretins including glucagon-like peptide-1 (GLP-1) and gastric inhibitory polypeptide (GIP) exert a bone protective and osteoanabolic effect as documented by lowered bone resorption, and increased bone mass (16–20). In line with the aforementioned preclinical data, *in-vitro* studies also suggest that activation of GIP and GLP-1 receptor promotes the osteoblast cell lines differentiation and bone formation (19, 21, 22). Dipeptidyl peptidase-4 inhibitors (DPP-4Is) are a type of anti-diabetic drug that protects bones by preventing incretin degradation (23, 24). Previous *in-vitro* and *in-vivo* studies corroborate that DPP-4 inhibitors (sitagliptin, vildagliptin, and teneligliptin) can potentiate bone formation and improve bone microarchitecture (25–31). Clinical studies and meta-analyses back up the above evidence of bone-protective effects, as evidenced by reduced fracture risk and improved bone metabolism (32–34).

Linagliptin is unique among DPP-4 inhibitors because of its added benefits, such as once-daily dosing, low drug interactions, and a non-renal route of administration, which eliminates the need for dose adjustments in the case of renal or hepatic impairment (35, 36). After alogliptin, linagliptin is the only DPP-4 inhibitor that has the highest selectivity for the DPP-4 enzyme (37). Linagliptin has already been reported to lower fracture risk in diabetic patients (34, 38, 39). A recent study also demonstrated its role in reducing bone fragility in obese mice (39). Metformin is one of the most prescribed drugs for the treatment of type 2 diabetes (40). Metformin reduces fracture risk and protects bone health as per several clinical, preclinical, and *in-vitro* studies (41–48). Currently, linagliptin is clinically prescribed either as a monotherapy in metformin intolerant patients or as a combination with metformin for better glycemic control (35, 49, 50). However, the role of the combination of linagliptin and metformin in type 2 diabetes-associated osteoporosis has not been investigated yet.

In the present work, we investigated the bone protective potential of linagliptin, metformin, and their combination for the treatment of diabetic osteoporosis in C57BL/6 mice. We induced diabetic osteoporosis in C57BL/6 mice by administering HFD (60%Kcal fat) as, among various models of type 2 diabetes, the excessive calorie-based HFD model is capable of developing phenotypes that mimic human pathophysiology (51).

## Materials And Methods

### Chemicals and Kits

Metformin and linagliptin were procured as gift samples from NishChem International Pvt Ltd, Mumbai, India. Biochemical kits of Alkaline Phosphatase (ALP), serum calcium, triglyceride, and cholesterol were purchased from Erba diagnostics. Enzyme-linked immunosorbent assay (ELISA) kits for Tumor Necrosis Factor-alpha (TNF-α), Interleukins-6 (IL-6), Interleukins (IL-β), and sclerostin were purchased from Krishgen biosystem, India. Osteocalcin and Bone morphogenetic protein- 2 (BMP-2) ELISA kits were purchased from Abbkine Inc. Mouse leptin ELISA kit was purchased from My Biosouce, Inc.

### Animals and Experimental Protocol

C57BL/6 male mice (7 weeks old) weighing 20-22g were procured from the central animal house facility (CAHF) of Jamia Hamdard. They were housed in the polypropylene cages under standard conditions (22 ± 2°C; 55% humidity) on a 12h light/dark cycle. All experiments were performed by following the guidelines of the committee for the purpose of control and supervision of experiments on animals (CPCSEA) and approved by Jamia Hamdard Institutional Animal Ethics Committee (approval no. 1512).

Mice were randomly divided into two groups and fed with either a normal pellet diet (ND) or a high-fat diet (HFD) for 18 weeks to induce diabetes. HFD containing 60% kcal fat, 25% protein & 17% carbohydrate was prepared in our laboratory following the previous method (52). The food intake and body weight were recorded at regular intervals of time. For the confirmation of diabetes, fasting blood glucose level (FBG) was weekly monitored using a glucometer by withdrawing blood from the tail vein.

**Figure 1:**
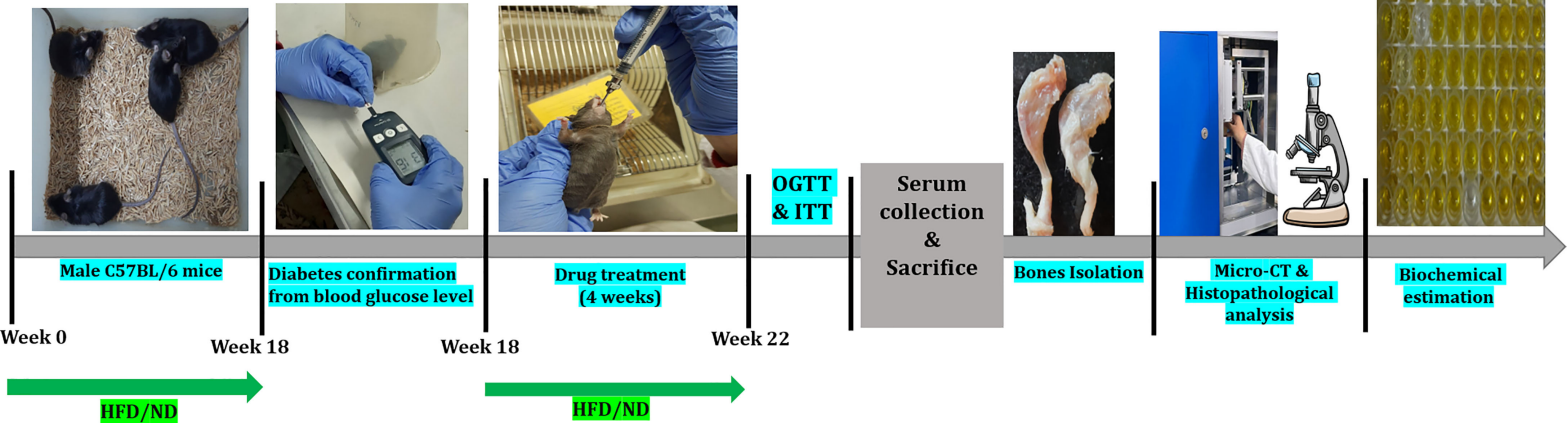
Schematic presentation of the experimental design of the study. HFD, High-fat diet; ND, Normal pellet diet; OGTT, Oral glucose tolerance test; ITT, Insulin tolerance test.

After 18 weeks of HFD feeding, a simple randomization technique was used to divide all HFD fed diabetic mice into following 4 groups (n=8 per group): HFD group; HFD+L group (Linagliptin 10mg/kg/day); HFD+M group (Metformin 150mg/kg/day); HFD+LM group (Combination of linagliptin 10mg/kg/day & metformin 150mg/kg/day). Doses of linagliptin and metformin were chosen according to the previous studies (53, 54). All drug treatments were dissolved in 0.5% CMC and given orally for the next 4 weeks. Mice in the control and HFD groups received an equal amount of 0.5% CMC orally for 4 weeks. A schematic representation of the experimental design is outlined in **Figure 1**. Based on the previous study (29), the sample size (n=8) was calculated by using the G Power 3.0.10 version software and the values obtained were as follows: effect size (d) = 1.97, α error probability = 0.05, power (1-β) = 0.95 and allocation ratio (N2/N1) = 1. Animal Research Reporting of *In-vivo* Experiments (ARRIVE) guidelines were followed for reporting all experimental data.

### *In-Vivo* Glucose and Insulin Tolerance Test

After 4 weeks of drug intervention, an oral glucose tolerance test (OGTT) was performed. For OGTT, mice were subjected to 12h of fasting, and baseline (0 min) blood glucose level was measured using a glucometer. Following this, 2g/kg of glucose (p.o.) was administered and a blood sample from the tail vein was collected at 15, 30, 60, 120, and 180 min accompanied by blood glucose measurements. After 6 days of OGTT, IGTT was performed on fasting mice by injecting insulin at the dose of 0.75U/kg, i.p. followed by blood glucose measurements at 0, 15, 30, 60, and 120 min of the time interval.

### Sample Preparation

At the end of the study, mice were anesthetized using CO2, and blood was collected from cardiac puncture. Serum was separated by centrifugation at 1500*g* for 15 min at 4°C. The collected serum was immediately stored at -80°C till further analysis. Mice were sacrificed to isolate both left-right femur and tibia which were cleaned and stored at -20°C till further scanning and assessment.

### Bone Micro-Architecture Analysis Using μCT

Micro-computed tomography (μCT) of excised left femur and tibia was performed using a Scanco micro-CT40 scanner (Scanco Medical AG, Bruettisellen, Switzerland). Scanning was performed according to the previously recommended guidelines (55). In brief, bones were scanned at 8 mm isotropic voxel size with a 2.55kVp filter and X-ray source intensity set as 55 kVp, 145 μA, and integration time of 200ms. A fixed threshold of 275 was used to extract the mineralized bone from the soft tissue and marrow phase. Reconstruction was performed on a total of 50 slices for trabecular and cortical bone indexes by using the software provided by Scanco. The distal femur and proximal tibia were selected for reconstruction because maximum degradation is observed in these regions. Ellipsoid contours were drawn using CT Analyzer in the selected trabecular and cortical regions of bone. For the trabecular analysis, the region of interest (ROI) was drawn at a total of 50 slices in the secondary spongiosa region located at 1.5 mm from the distal border of the growth plate for femoral bones and proximally for tibia bones, eliminating all primary spongiosa and cortical bone. To analyze cortical indexes, a scan of 50 slices at the femoral and tibial mid diaphysis was chosen to perform quantification using Scanco software. The threshold of 275 was selected by positioning the threshold lines to be clear of noise fluctuation near the central region (vertically) of the display (56). Bone micro-architecture parameters recorded were: Bone Volume Fraction (BV/TV, %), Trabecular number (Tb. N, 1/mm), Trabecular thickness (Tb. Th, mm), Trabecular separation (Tb. Sp, mm), Connectivity density (Conn. D, 1/mm³), Structural model Index (SMI), Total cross-sectional tissue area (Tt. Ar, mm^2^), Cortical Bone area (Ct. Ar, mm^2^) and Cortical Thickness (Ct. Th, mm) and Bone mineral density (BMD, g/cm^3^).

### Biochemical Analysis

Serum triglyceride, total cholesterol, and calcium were quantified using the commercial diagnostic kits while serum TNF-α, IL-6, IL-β, and leptin were measured using mouse ELISA kits following the manufacturer’s instructions.

Femur bones of the right leg were crushed using liquid nitrogen and the bone homogenate was prepared by the HCl-ABC method, following the standard protocol (57). Bone homogenates were used for the assessment of the following bone turnover markers: ALP was estimated by a commercial diagnostic kit, and tartrate−resistant acid phosphatase (TRAP5b) was quantified using the previously standardized method (58), osteocalcin, BMP-2, and sclerostin were assessed using an ELISA assay kit.

### Bone Histopathological Analysis

Left femurs of each group were fixed in 4% formalin and demineralized in 10% ethylene diamine tetra acetic acid (EDTA) for 14 days. Then, tissues were dehydrated, cleared, and embedded in paraffin blocks. With the help of a microtome, the processed tissues were cut into the longitudinal section of about 5 μm thickness, placed on the glass slide, and stained with hematoxylin and eosin (H & E) stain to observe the bone trabecular region and population of bone cells i.e., osteoblast, osteoclast, and osteocytes in different treatment groups. All slides were viewed under the light microscope at 4X and 40X magnification.

### Immunohistochemistry Analysis for BMP-2 Expression in Bone Sections

Besides ELISA-based evaluation of BMP-2 protein, the immunohistochemistry (IHC) of the femur trabeculae section of bone was performed for further analysis of BMP-2 expression in treatment groups. Briefly, slides were deparaffinized, rehydrated, and washed. To avoid the non-specific background staining due to endogenous peroxidase, slides were incubated in a 4% hydrogen peroxide block followed by washing and incubation. Then, sections were exposed to the primary antibody of BMP-2 at 4°C overnight. Thereafter, slides were washed with saline and stained with DAB reagent followed by counterstaining with hematoxylin. The positively expressed BMP-2 protein, identified by brown stain was observed by the light microscope.

### Statistical Analysis

All data are numerically expressed as Mean± SD. Two-way ANOVA was used for statistical analysis of parameters like food intake, body weight, and fasting blood glucose level. When ANOVA revealed significant differences, the Bonferroni *post hoc* test was applied for comparisons between means. All other data were analyzed using one-way ANOVA followed by Tukey’s multiple comparisons. p<0.05 values are considered statistically significant. Analysis was performed using the software GraphPad Prism, version 8.0.2.

## Results

### Influence of Linagliptin, Metformin and Their Combination on Food Intake, Body Weight, and Fasting Blood Glucose of HFD Fed Mice

**Figure 2:**
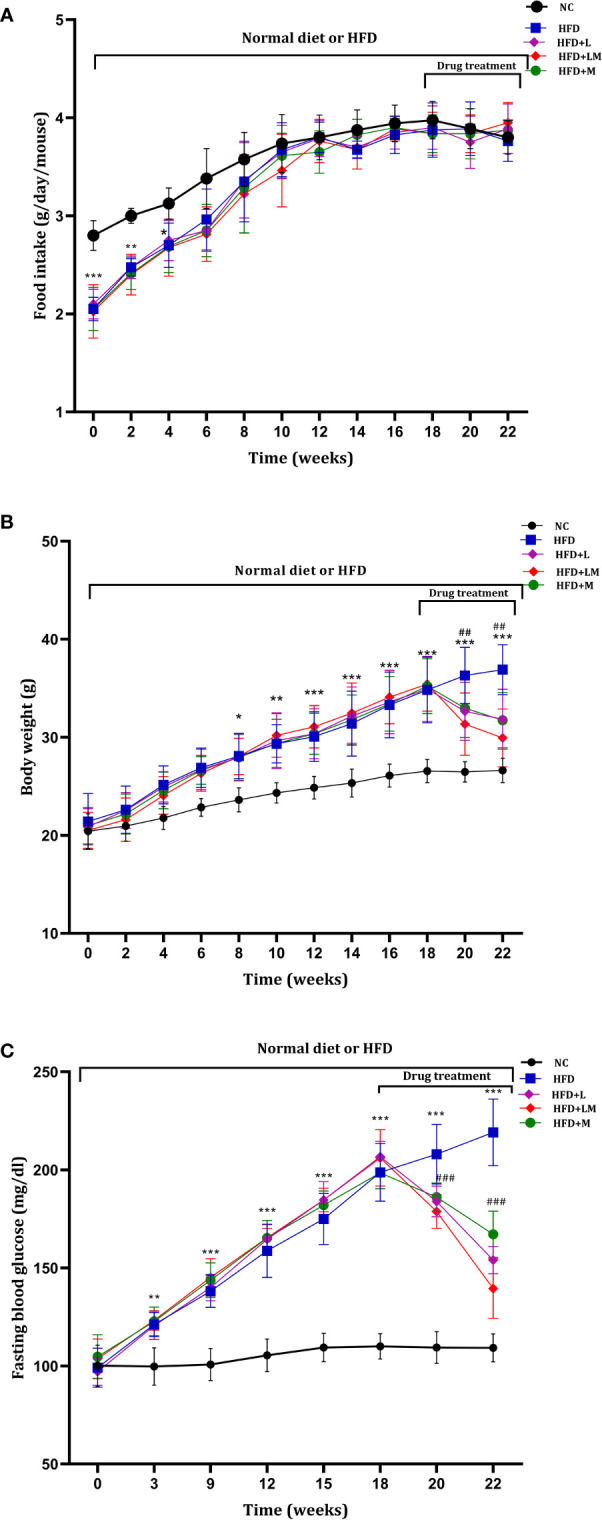
Weekly changes in **(A)** food intake, **(B)** body weight, and **(C)** fasting blood glucose among different treatment groups. Mice were fed a normal pellet diet (ND) or HFD for 22 weeks and drugs were administered from 18^th^ to 22^nd^ week (n=8) in all treatment groups. All data are presented as mean ± SD. ***p<0.001, **p<0.01 and *p<0.05 when compared with NC group, ^###^p<0.001 and ^##^p<0.01 when compared with HFD group. Significance is statistically analyzed by two-way ANOVA followed by the Bonferroni *post hoc* test. NC, Normal control (0.5% CMC); HFD, High fat diet (0.5% CMC); L, Linagliptin (10mg/kg); M, Metformin (150 mg/kg); LM, Linagliptin & Metformin combination (10mg/kg & 150mg/kg).

Mice on HFD showed significantly reduced food intake for the initial 4 weeks which started resuming to normal from the 6^th^ week onwards. HFD fed animals treated with linagliptin, metformin, and their combination from 18^th^ to 22^nd^ week did not alter the food intake **(** **Figure 2A** **).**

HFD-fed mice demonstrated a significant increase in body weight from the 8^th^ week onwards and continued till the end of the study. Treatment with linagliptin, metformin, and their combination from 18^th^-22^nd^ week resulted in a significant reduction in HFD-induced increase in body weight (**Figure 2B** **)**. This signifies the weight-reducing potential of linagliptin, metformin, and their combination.

As compared to the control group, from the 3^rd^ week of HFD feeding, mice exhibited a significant increase in blood glucose, which gradually increased till the end of the study. Treatment with linagliptin, metformin, and their combination (18^th^ to 22^nd^ week) presented a significant reduction in HFD escalated blood glucose (**Figure 2C** **).**

### Linagliptin, Metformin, and Their Combination Improved HFD Altered OGTT and ITT

**Table 1** depicts the result of the Oral glucose tolerance test (OGTT) and Insulin tolerance test (ITT), performed after the completion of 22 weeks of study. OGTT results suggest that in comparison with normal mice, oral glucose loading in HFD-fed mice resulted in hyperglycemia and glucose intolerance. Treatment with linagliptin, metformin, and their combination for 4 weeks exhibited improvement in glucose tolerance Similarly, ITT results delineate that HFD feeding caused high insulin resistance which is indicated by a gradual decline in blood glucose levels after injection of insulin at a dose of 0.75U/kg. Treatment with linagliptin, metformin, and their combination resulted in the rapid reduction of blood glucose levels, which is indicative of lower insulin resistance and attenuation of diabetes.

**Table 1:** Effect of linagliptin, metformin, and their combination on Oral glucose tolerance test (OGTT) and Insulin tolerance test (ITT).

Time (min)	Blood glucose (mg/dl)				
	NC	HFD	HFD+L	HFD+M	HFD+LM
**OGTT**					
0	109.4 ± 8.14	212 ± 25.28***	151.8 ± 7.63^###^	162.2 ± 15.37^#^	140 ± 19.46^###^
15	183.1 ± 15.02	358.6 ± 35.31***	214.6 ± 17.52^###^	224 ± 36.92^###^	190.2 ± 13.58^###^
30	209.4 ± 1.63	406.5 ± 46.38***	242.4 ± 12.06^###^	213.2 ± 24.03^###^	182.8 ± 32.37^###^
60	161 ± 15.33	270.8 ± 73.83***	180.8 ± 11.84^###^	160.5 ± 17.72^###^	167.8 ± 18.41^###^
120	132.3 ± 21.10	200 ± 42.31***	137.2 ± 20.10^###^	127.1 ± 19.05^###^	129.7 ± 12.12^###^
180	120 ± 9.62	179.6 ± 32.26***	127.8 ± 14.31^##^	128.6 ± 28.33^##^	122.2 ± 11.45^##^
**ITT**	**Blood glucose (mg/dl)**				
0	112.2± 7.92	228 ± 10.66***	165.6 ± 8.7^###^	169.6 ± 11.47^###^	166.1 ± 12.29^###^
15	89.3 ± 7.87	211.3 ± 12.6***	114 ± 8.31^###^	118.6 ± 11.38^###^	109.1 ± 16.53^###^
30	74.8 ± 5.98	180.7± 7.74***	81 ± 6.02^###^	83.750 ± 6.92^###^	72.7 ± 10.02^###^
60	52.7 ± 4.89	172.1 ± 7.21***	54.5 ± 9.56^###^	52.000 ± 8.41^###^	43 ± 5.37^###^
120	57.1 ± 8.99	182.8 ± 7.35***	50 ± 7.72^###^	46.500 ± 6.09^###^	50.7 ± 6.75^###^
180	67.3 ± 13.26	191.2 ± 6.27***	50.6 ± 5.70^###^	47.625 ± 7.53^###^	53.8 ± 9.44^###^

Mice were fed a normal pellet diet (ND) or HFD for 22 weeks and the drug was given from the 18^th^ week to 22^nd^ weeks in all treatment groups. All data are presented as mean ± SD (n=8). ***p<0.001 when compared with NC group, ^###^p<0.001 and ^##^p<0.01, ^#^p<0.05 when compared with HFD group. Significance is statistically analyzed by two-way ANOVA followed by the Bonferroni post hoc test. NC, Normal control (0.5% CMC); HFD, High fat diet (0.5% CMC); L, Linagliptin (10mg/kg); M, Metformin (150 mg/kg); LM, Linagliptin & Metformin combination (10mg/kg & 150mg/kg).

### Changes in Leptin, Triglycerides, and Total Cholesterol Levels

**Figure 3:**
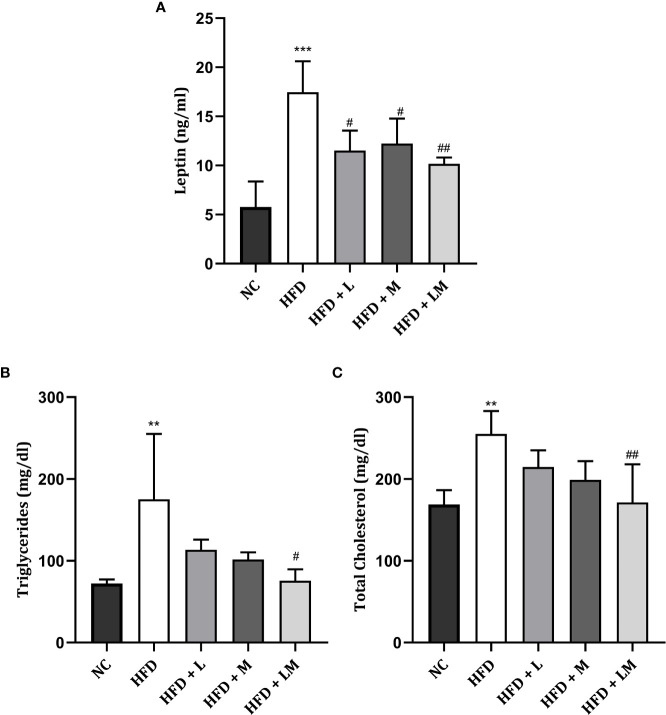
Effect of linagliptin, metformin, and their combination on Leptin **(A)**, Triglycerides **(B)**, and Cholesterol **(C)**. Mice were fed a normal pellet diet (ND) or HFD for 22 weeks and the drug was given from the 18^th^ week to the 22^nd^ week in all treatment groups. All data are presented as mean ± SD (n=4). ***p<0.001 and **p<0.01 when compared with NC group, ^##^p<0.01 and ^#^p<0.05 when compared with HFD group. Significance is statistically analyzed by one-way ANOVA followed by Tukey’s multiple comparisons. NC, Normal control (0.5% CMC); HFD, High fat diet (0.5% CMC); L, Linagliptin (10mg/kg); M, Metformin (150 mg/kg); LM, Linagliptin & Metformin combination (10mg/kg & 150mg/kg).

As portrayed in **Figure 3A**, serum leptin was significantly increased in HFD-fed mice (p<0.001). Treatment with linagliptin, metformin, and their combination showed a significant reduction in the elevated leptin levels with the maximum reduction observed in the combination group (p<0.01).

The lipid profile was unveiled by estimating triglyceride (**Figure 3B** **)** and total cholesterol (**Figure 3C** **)** levels. HFD feeding resulted in a significant increase in triglyceride and total cholesterol levels (p<0.01). Treatment with the combination of linagliptin and metformin resulted in a significant reduction in HFD-associated increase in triglyceride and total cholesterol levels. Though linagliptin and metformin alone also reduced triglycerides and cholesterol levels, results were not found to be significant.

### Linagliptin in Combination With Metformin Improved HFD-Impaired Trabecular and Cortical Bone Micro-Architecture

**Figure 4:**
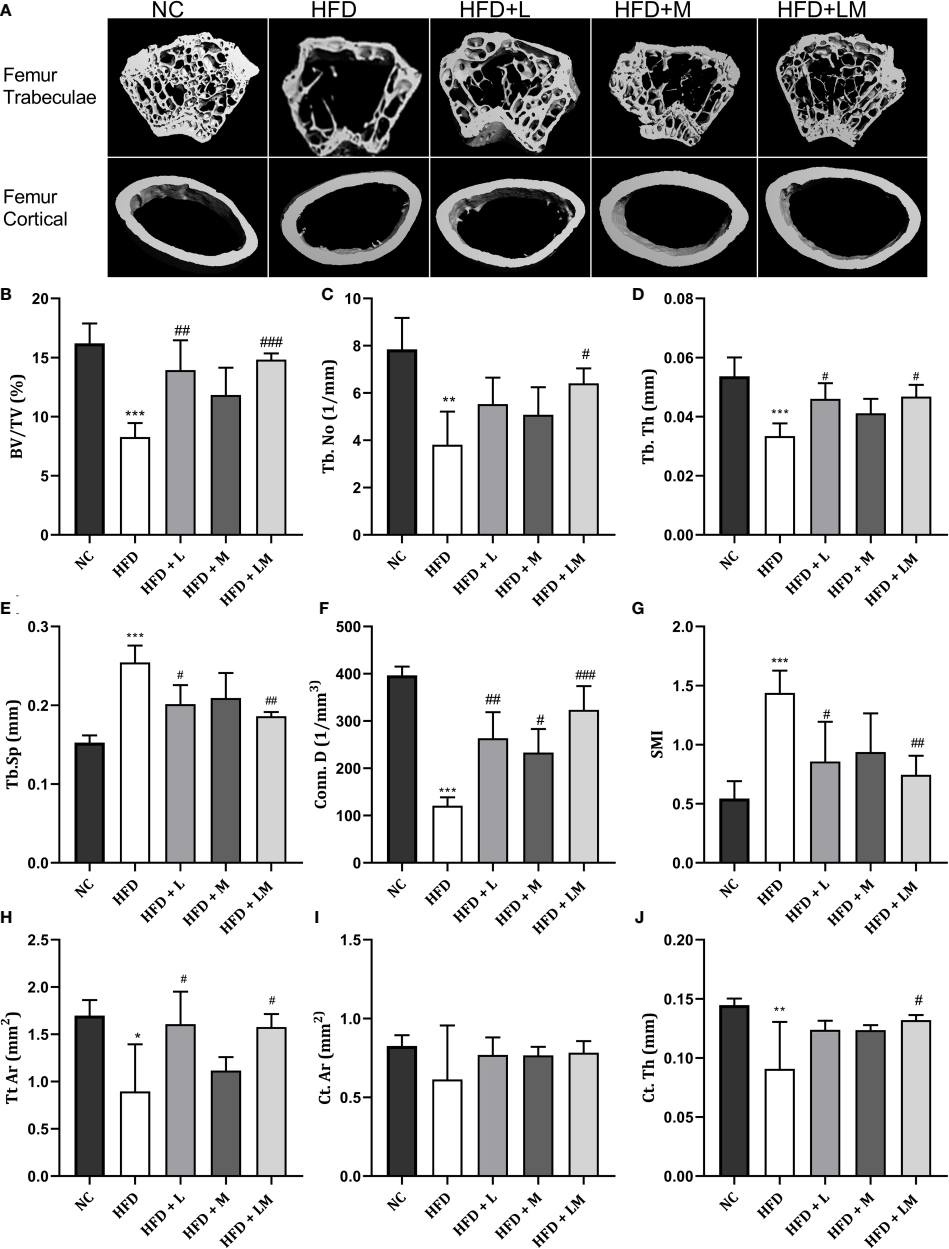
Effect of linagliptin, metformin, and their combination on HFD impaired trabecular and cortical bone micro-architecture of the femur using μCT. Representative 3D micro-CT images of the distal femur trabecular and mid diaphysis cortical region of different treatment groups **(A)**; Quantification of distal femur trabecular bone parameters i.e., Bone volume/trabecular volume (BV/TV), Trabecular number (Tb. N), Trabecular thickness (Tb. Th), Trabecular separation (Tb. Sp), Connectivity density (Conn. D) and Structural model Index (SMI) **(B–G)**; Quantification of femur mid diaphysis cortical parameters i.e., Total cross-sectional tissue area (Tt. Ar), Cortical Bone area (Ct. Ar) and Cortical Thickness (Cortical. Th) **(H–J)**. All data are presented as mean ± SD (n=4). ***p<0.001, **p<0.01 and *p<0.05 when compared with NC group; ^###^p<0.001, p<0.01 ^##^p<0.01 and ^#^p<0.05 when compared with HFD group. Significance is statistically analyzed by one way-ANOVA followed by Tukey’s multiple comparisons. NC, Normal control (0.5% CMC); HFD, High fat diet (0.5% CMC); L, Linagliptin (10mg/kg); M, Metformin (150 mg/kg); LM, Linagliptin & Metformin combination (10mg/kg & 150mg/kg).

**Figure 5:**
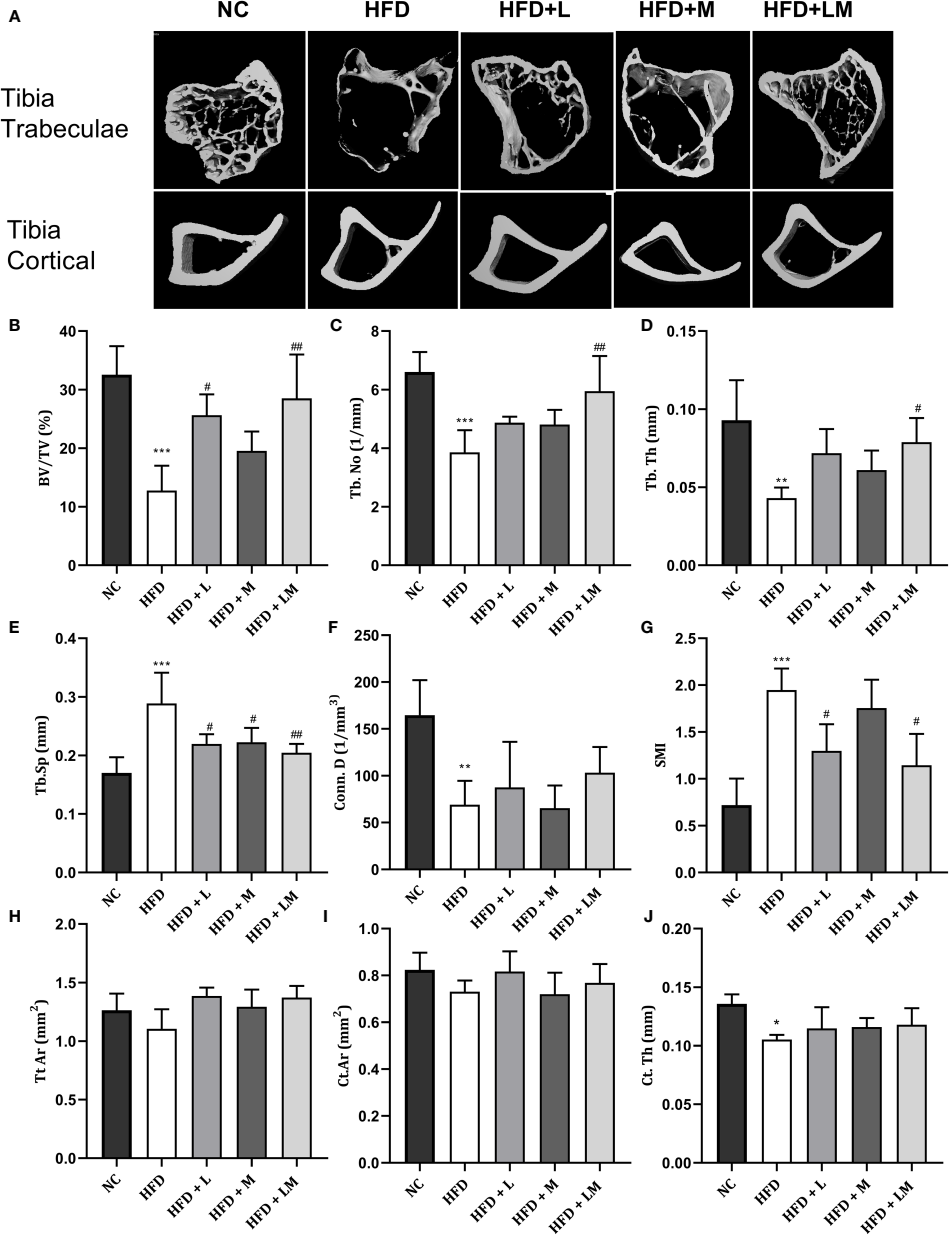
Effect of linagliptin, metformin, and their combination on HFD impaired tibial trabecular and cortical bone micro-architecture using μCT. Representative 3D micro-CT images of the proximal tibia trabecular and mid diaphysis cortical region **(A)**; Quantification of proximal tibia trabecular bone parameters i.e., Bone volume/trabecular volume (BV/TV), Trabecular number (Tb. N), Trabecular thickness (Tb. Th), Trabecular separation (Tb. Sp), Connectivity density (Conn. D) and Structural model Index (SMI) **(B–G)**; Quantification of tibia mid diaphysis cortical parameters i.e., Total cross-sectional tissue area (Tt. Ar), Cortical Bone area (Ct. Ar) and Cortical Thickness (Cortical. Th) **(H–J)**. All data are presented as mean ± SD (n=4). ***p<0.001, **p<0.01 and *p<0.05 when compared with NC group; ^##^p<0.01 and ^#^p<0.05 when compared with HFD group. Significance is statistically analyzed by one way-ANOVA followed by Tukey’s multiple comparisons. NC, Normal control (0.5% CMC); HFD, High fat diet (0.5% CMC); L, Linagliptin (10mg/kg); M, Metformin (150 mg/kg); LM, Linagliptin & Metformin combination (10mg/kg & 150mg/kg).

HFD-fed mice showed a significant reduction in BV/TV, Tb. No, Tb, Th, Conn. D, and a significant increase in Tb.Sp and SMI of distal femur trabeculae. Treatment with linagliptin alone, and in combination with metformin reversed the HFD-induced bone damage as indicated by an increase in BV/TV, Tb, Th, and Conn. D and a significant reduction in Tb.Sp and SMI. Femur cortical indices revealed that relative to the control group, HFD-fed mice showed a reduction in Tt. Ar and Ct.Th while Ct. Ar remained unchanged. Mice treated with the combination of linagliptin and metformin illustrated a significant increase in Tt. Ar and Ct. Th while linagliptin increased Tt. Ar only. Although treatment with metformin also revealed slight improvement in HFD-induced bone damage, the results observed were not found to be significant (**Figures 4A** **–J** **)**.

the Proximal tibia trabecular micro-CT indices of HFD-fed mice also revealed a similar pattern as the distal femur, however, cortical indices results were not as prominent as observed in the femur. Likewise, treatment with linagliptin and its combination with metformin also exhibited a similar pattern as seen with the femur (**Figures 5A** **–J** **).** The above results demonstrate that the combination of linagliptin and metformin shows the maximum bone protective effect, which is followed by linagliptin treatment.

### Linagliptin, Metformin, and Their Combination Increased BMD in HFD Fed Mice

**Figure 6:**
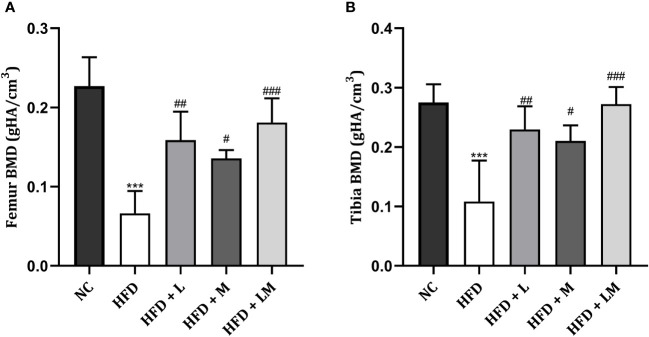
Effect of linagliptin, metformin and their combination on BMD in HFD fed mice. BMD of distal femur **(A)** and proximal tibia **(B)** regions of bone was evaluated using micro-CT. All data is presented as mean ± SD (n=4). ***p<0.001 when compared with NC group, ###p<0.001, ##p<0.01, and #p<0.05 when compared with HFD group. Significance is statistically analyzed by one-way ANOVA followed by Tukey’s multiple comparison. NC, Normal control (0.5% CMC); HFD, High fat diet (0.5% CMC); L, Linagliptin (10mg/kg); M, Metformin (150 mg/kg); LM, Linagliptin & Metformin combination (10mg/kg & 150mg/kg).

HFD feeding reduced the BMD in the distal femur and proximal tibia region (p<0.001) as evaluated by micro-CT. Treatment with linagliptin, metformin, and their combination significantly increased the HFD altered BMD in both distal femur and proximal tibia. A combination of linagliptin and metformin showed maximum improvement in BMD (p<0.001) as compared to other treatment groups **(** **Figures 6A, B** **)**.

### Impact of Linagliptin, Metformin, and Their Combination on HFD Altered Bone Turnover Biomarkers

**Figure 7:**
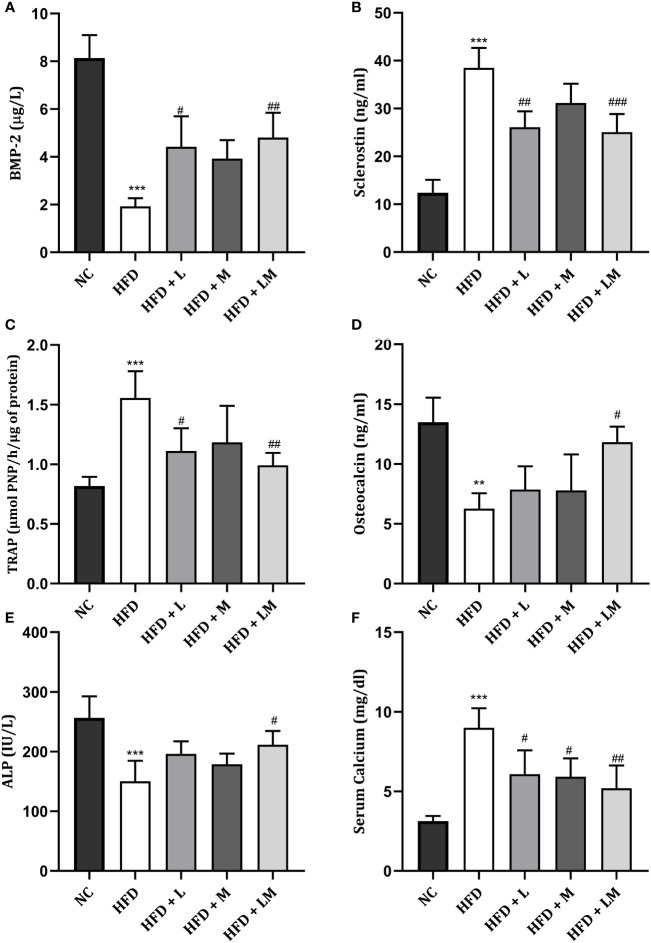
Effect of linagliptin, metformin and their combination on bone turnover biomarkers. **(A)** Bone morphogenetic protein-2 (BMP-2), **(B)** Sclerostin, **(C)** Tartrate−resistant acid phosphatase (TRAP), **(D)** Osteocalcin, **(E)** Bone specific Alkaline phosphatase (ALP), **(F)** Serum Calcium. All data is presented as mean ± SD (n=4). ***p<0.001 and **p<0.01 when compared with normal control; ^###^p<0.001, ^##^p<0.01 and ^#^p<0.05 when compared with HFD group. Significance is statistically analyzed by one-way ANOVA followed by Tukey’s multiple comparison. NC, Normal control (0.5% CMC); HFD, High fat diet (0.5% CMC); L, Linagliptin (10mg/kg); M, Metformin (150 mg/kg); LM, Linagliptin & Metformin combination (10mg/kg & 150mg/kg).

HFD-fed mice demonstrated a significant reduction in ALP, osteocalcin, and BMP-2 while a significant increase in serum calcium, TRAP, and sclerostin levels. Treatment with the combination of linagliptin and metformin reverted these changes. Though linagliptin also increased BMP-2 (p<0.05) and reduced TRAP, sclerostin, and serum calcium levels, there were no significant changes observed in ALP and osteocalcin. Though metformin also showed a tendency to reverse the HFD altered bone turnover markers, the significant changes were observed only with serum calcium (**Figures 7A** **–F** **)**.

### Linagliptin, Metformin, and Their Combination Reduced HFD Triggered Pro-Inflammatory Cytokines

**Figure 8:**
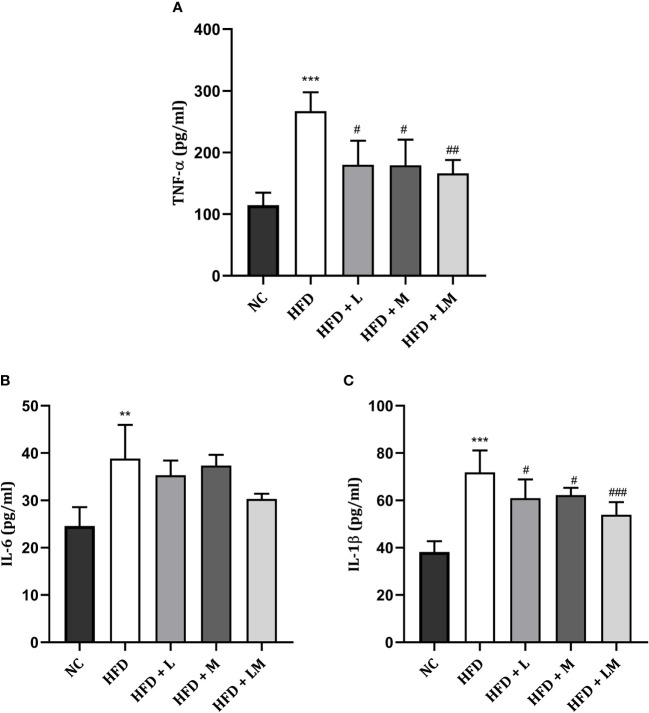
Effect of linagliptin, metformin and their combination on pro-inflammatory cytokines **(A)** TNF-α, **(B)** IL-6, **(C)** IL-1β. All data is presented as mean ± SD (n=4). ***p<0.001 and **p<0.01 when compared with NC group; ^###^p<0.001, ^##^p<0.01 and ^#^p<0.05 when compared with HFD group. Significance is statistically analyzed by one-way ANOVA followed by Tukey’s multiple comparison. NC, Normal control (0.5% CMC); HFD, High fat diet (0.5% CMC); L, Linagliptin (10mg/kg); M, Metformin (150 mg/kg); LM, Linagliptin & Metformin combination (10mg/kg & 150mg/kg).

**Figure 8** shows the level of pro-inflammatory cytokines i.e., TNF-α, IL-6, and IL-1β in all treatment groups which were significantly increased in HFD-fed mice. Drug treatment with linagliptin, metformin, and their combination significantly reduced the HFD-triggered TNF- α and IL-1β level with the maximum effect observed in the combination group (p<0.01). No significant differences were observed in IL-6 levels in either drug-treated or the combination group.

### Bone Histopathology of HFD Fed Mice Treated With Linagliptin, Metformin, and Their Combination

4X images demonstrate that the trabecular bone in the control group appeared to be thicker and regularly arranged, while HFD feeding damaged the bone histology as evidenced by thinner, irregular, and ruptured appearance of bone. Treatment with the combination of linagliptin and metformin resulted in improved bone thickness and arrangement.

**Figure 9:**
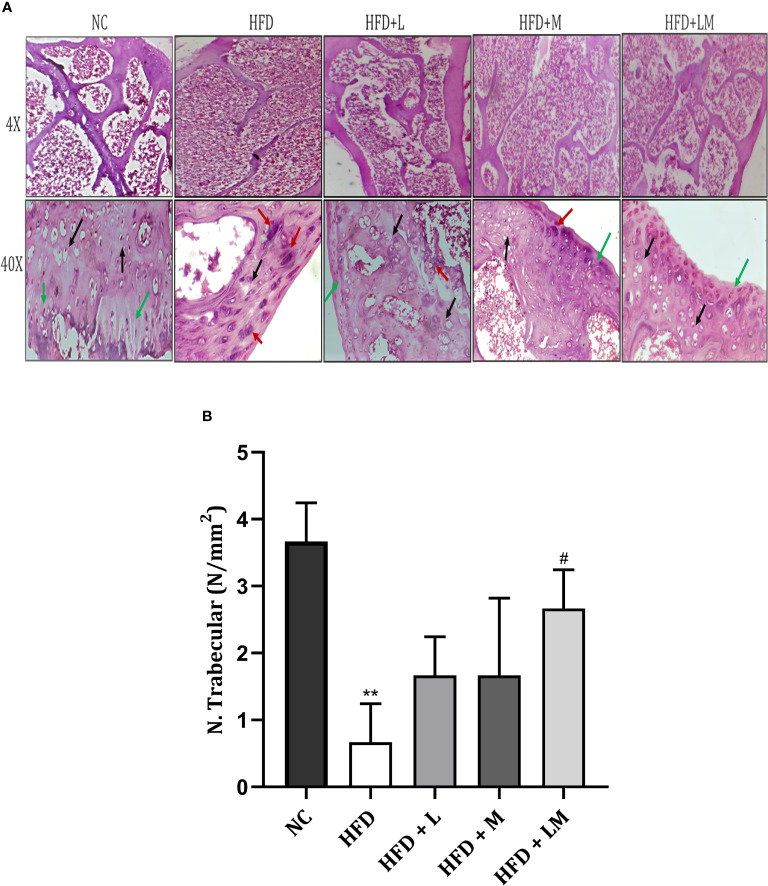
Effect of linagliptin, metformin, and their combination on bone histopathology. **(A)** Representative images of H & E staining of femur trabecular bone at 4X and 40X, **(B)** trabecular number per square millimeter (N/mm2). All data are presented as mean ± SD (n=3). **p<0.01 when compared with NC group; #p<0.05 when compared with HFD group. Significance is statistically analyzed by one-way ANOVA followed by Tukey’s multiple comparisons. NC, Normal control (0.5% CMC); HFD, High fat diet (0.5% CMC); L, Linagliptin (10mg/kg); M, Metformin (150 mg/kg); LM, Linagliptin & Metformin combination (10mg/kg & 150mg/kg).

Histopathological images viewed at 40X reveal the appearance of different bone cells i.e., osteoblast (green arrow), osteocytes (black arrow), and osteoclasts (red arrow). Qualitative observation of HFD-fed mice demonstrated a greater number of osteoclasts and a smaller number of osteoblasts and osteocytes indicative of severe bone damage. Treatment with linagliptin, metformin, and their combination reversed these changes as visualized by an increased population of osteoblasts and osteocytes and reduced osteoclasts cells **(** **Figure 9A** **).**

Quantitative analysis of the trabecular number per square millimeter (N.Trabecular/mm^2^) presented in **Figure 9B**, showed a reduction in trabecular number in the HFD-fed group. (p<0.01). Mice treated with a combination of linagliptin, and metformin presented an increased trabecular number (p<0.05) while treatment with either linagliptin or metformin did not show any significant effect.

### Linagliptin and Its Combination With Metformin Showed Higher Expression of BMP-2 in Bone Immunohistochemistry

**Figure 10:**
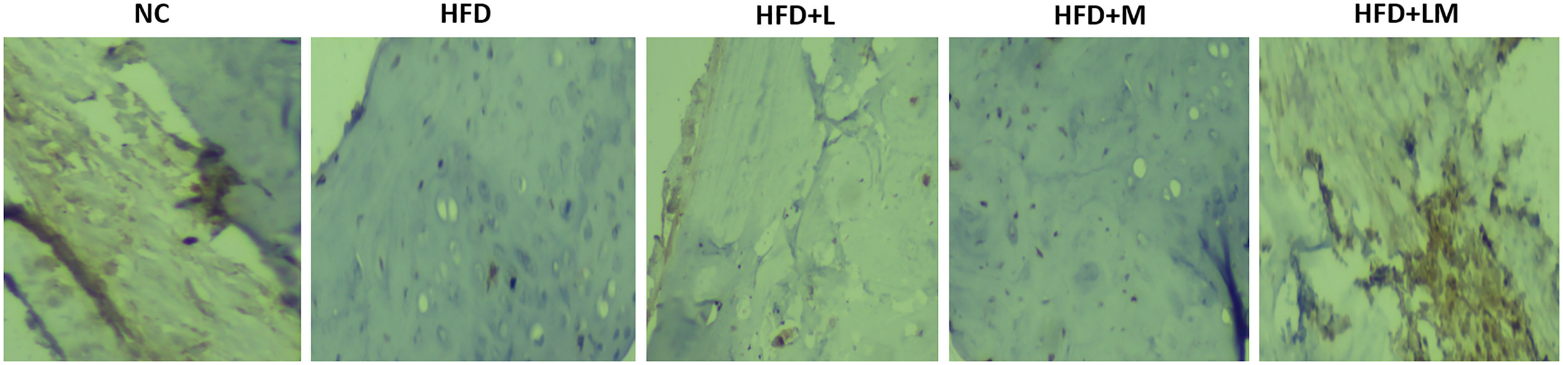
Effect of linagliptin, metformin, and their combination on immunohistochemistry analysis of BMP-2 protein. Representative images of IHC stained femur trabecular bone at 40X. Cells with negative expression of BMP-2 protein are stained blue, while cells with positive BMP-2 expression is seen by brown staining. NC, Normal control (0.5% CMC); HFD, High fat diet (0.5% CMC); L, Linagliptin (10mg/kg); M, Metformin (150 mg/kg); LM, Linagliptin & Metformin combination (10mg/kg & 150mg/kg).

Visual analysis of IHC stained sections of femur trabeculae matrix of control mice represents a significantly stronger expression of positive immuno-staining cells which indicates the abundant number of bones forming BMP-2 protein. As compared to the control group, HFD feeding demonstrated the lower signaling of BMP-2 positive expressing cells. Treatment with the combination of linagliptin and metformin showed stronger positive expression of BMP-2, as indicated by the high population of immune positive cells. However, linagliptin and metformin alone treatment showed fewer BMP-2 immuno-positive cells **(** **Figure 10** **)**.

## Discussion

Bone disorders including osteoporosis are the serious complications of type 2 diabetes mellitus, which causes detrimental effects on the quality, strength, and architecture of bone (59, 60). DPP-4 inhibitor in combination with metformin has been reported to lower the incidence of fracture risk and improve bone health in diabetic patients (61, 62). Linagliptin, one of the highly potent DPP-4 inhibitors, has been clinically proven to reduce fracture risk in diabetic patients (34, 38). However, to date, its potential to treat diabetic osteoporosis when combined with metformin was not explored. This is particularly important as DPP-4 inhibitor in combination with metformin is currently the most widely prescribed combination for type 2 diabetes, owing to additional benefits such as increased efficacy, tolerability, and lowered risk of hypoglycemia (63). In addition, the combination results in higher bone protective incretin levels such as GLP-1 in type-2 diabetes patients as compared to the DPP-4 inhibitor alone (24, 64). Here, we demonstrated for the first time that treatment with linagliptin and its combination with metformin can prevent diabetic osteoporosis through modulating BMP-2 and sclerostin in the high-fat diet fed C57BL/6 mice. The combination successfully prevented HFD-associated impaired bone architecture, BMD, and bone histopathology, and favorably modulated bone turnover biomarkers such as BMP-2, sclerostin, ALP, osteocalcin, TRAP, and serum calcium. Additionally, both drugs and their combination reversed HFD-related diabetes and metabolic abnormalities such as increased body weight, FBG, leptin, cholesterol, triglycerides, proinflammatory cytokines, impaired glucose, and insulin tolerance.

We used a high-fat diet model to induce diabetes and related osteoporosis symptoms in C57BL/6 mice. The primary changes that develop in rodents fed with HFD are weight gain and adipose tissue production which eventually develop into metabolic abnormalities including hyperglycemia and hyperlipidemia (65). In our study, after eight weeks on the HFD, the body weight of mice steadily increased followed by hyperglycemia, hyperlipidemia, and impaired glucose and insulin tolerance, which is in accordance with the previous *in-vivo* studies (66–71). Poor glycemic control is a significant risk factor for fractures in diabetic patients (72). In our study, 22 weeks of HFD feeding caused profound damage to the trabecular region of the distal femur and proximal tibia, as indicated by altered bone microarchitecture and is in line with other *in-vivo* studies (56, 66, 67, 73). We also observed altered microarchitecture in the mid diaphysis cortical region of the femur and tibial bone following HFD. A recent study by Dai et al. showed that 16 weeks of HFD feeding in rodents causes trabecular bone loss but more than 16 weeks of HFD feeding might result in cortical bone damage (74) as observed by us. This indicates that HFD feeding for a longer duration is crucial for cortical damage, as also evidenced in a study by Mansur and co-workers (75). Further, in agreement with the previous *in-vivo* studies, we also demonstrated a significant reduction in trabecular BMD of the distal femur and proximal tibia region (76–78). HFD feeding also modulated the bone turnover biomarkers as indicated by the reduced bone ALP, osteocalcin, and BMP-2 levels, as well as increased, sclerostin, TRAP, and serum calcium. These observations are in consistency with the earlier *in-vitro* and preclinical findings, which manifest that diabetes linked hyperglycemia negatively impact the bone turnover biomarkers (66, 73, 77). Thus, HFD-associated hyperglycemia, impaired glucose and insulin tolerance along with alterations in bone microarchitecture and turnover markers as observed in our study confirmed the development of diabetic osteoporosis in C57BL/6 mice.

Previously, sitagliptin (a DPP-4 inhibitor) is reported to show bone protective and osteogenic effects in different animal models including streptozotocin-induced diabetic rat model, ovariectomized mice model, and HFD model (25–27, 31). Another DPP-4 inhibitor, vildagliptin, also improved the bone architecture by inhibiting bone resorption in Zucker diabetic fatty rat model (29). However, to the best of our knowledge, only a single study reported the effects of linagliptin to reduce bone fragility in genetically obese mice (39). The present study is the first to report the protective effects of linagliptin in an animal model of HFD-induced diabetic osteoporosis. We observed that linagliptin improved HFD-induced altered femur trabecular architecture and increased trabecular BMD in the distal femur and proximal tibia in line with the previous *in-vitro, in-vivo*, and clinical studies on other DPP-4 inhibitors (31, 34, 38). In addition, it improved HFD-altered bone turnover biomarkers and demonstrated a rise in BMP-2. Further, linagliptin also reduced sclerostin, TRAP, and serum calcium. In a previous study, it was reported that vildagliptin lowers the levels of sclerostin and TRAP in the Zucker diabetic fatty rat model (29) and our results are in agreement.

We observed that treatment with metformin improved the HFD modified trabecular BMD of the distal femur and proximal tibia region in agreement with the previous studies that indicate that metformin treatment improves BMD in mice (45, 54). However, we noted that treatment with metformin at the dose of 150mg/kg did not show any significant improvement in HFD altered bone architecture parameters and bone turnover biomarkers except for reduced serum calcium. Similar to our study, another study reported that at a lower dose of metformin i.e. 100mg/kg, it neither improves bone mass nor produces an osteogenic effect in C57BL/6 mice (79). Thus, working with different doses of metformin would be beneficial in the future to explore the effect of varying doses of metformin on diabetic osteoporosis in mice.

The combined treatment with linagliptin and metformin showed remarkable improvement in HFD-associated trabecular bone parameters of the distal femur and proximal tibia region. On bone turnover markers, the combined treatment resulted in an elevation of the levels of bone formation biomarkers i.e. bone BMP-2, ALP, and osteocalcin. These findings are consistent with the previous *in-vitro* reports which suggest that metformin and DPP-4 inhibitors (trelagliptin & anagliptin) increase bone formation biomarkers such as BMP-2, ALP, and osteocalcin in mouse osteoblastic MC3T3-E1 cell lines (28, 44, 80, 81).

Bone morphogenetic protein-2 (BMP-2) is an important bone turnover biomarker that regulates bone remodeling by regulating the differentiation of bone marrow mesenchymal cells and osteoprogenitors into osteoblasts (9, 82). An *in-vitro* study has reported that hyperglycemia results in reduced levels of BMP-2 in the bone mesenchymal stem cells (12). A clinical study also reported that the plasma levels of BMP-2 were higher in diabetic patients as compared to non-diabetic individuals, which might be indicative of the low BMP-2 concentrations in bone cells (83). In our study too, we observed that HFD-induced diabetes resulted in reduced bone BMP-2 levels which were reverted by linagliptin and its combination with metformin. It has been reported that metformin causes osteoblast differentiation by activating the AMPK signaling pathway and increasing BMP-2 expression which could be responsible for its bone protective effects (44). However, we could not find any significant effect of metformin on BMP-2 levels though its combination with linagliptin resulted in higher BMP-2 levels indicating that the pathway other than AMPK might be responsible for the protective effects of linagliptin and its combination. Stronger expression of BMP-2 immuno-positive cells in the bone sections of mice treated with a combination of linagliptin and metformin further confirms the involvement of the BMP-2 pathway.

Another important bone-turnover biomarker, sclerostin, which acts as a negative regulator of bone formation is identified as a strong inhibitor of the Wnt signaling pathway and bone formation (10, 11, 84). Diabetes is one of the responsible factors in regulating sclerostin concentration and is proven by a previous *in-vitro* study that reports that hyperglycemia results in sclerostin overexpression in murine cell lines (13). Clinically too, it was confirmed that circulating sclerostin levels are high in type 2 diabetic patients (14). Hence, blocking sclerostin can theoretically activate the Wnt pathway which promotes higher bone formation and lower bone resorption. We observed that linagliptin reduced sclerostin levels in congruity with past *in-vivo* studies which indicate that DPP-4 inhibitor vildagliptin and sitagliptin inhibit sclerostin levels (29, 85). Metformin, however, did not show any effect on sclerostin levels in our study. Further, the combination resulted in a higher reduction in sclerostin levels as compared to linagliptin alone.

**Figure 11:**
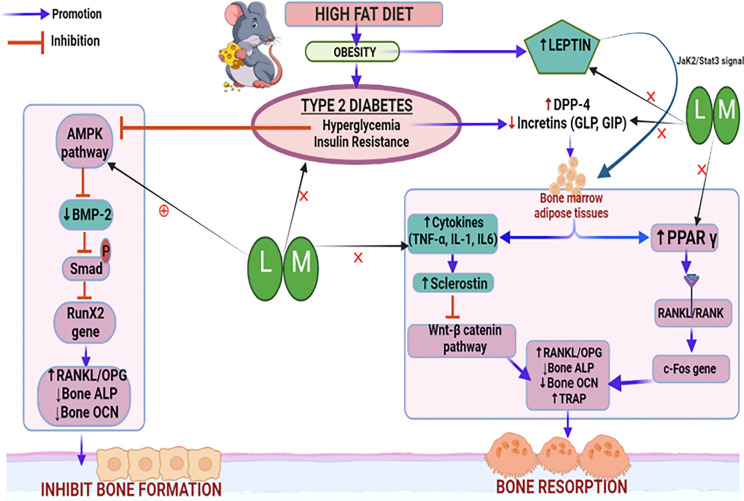
Schematic representation of the probable mechanistic explanation behind the bone protective effect of linagliptin and metformin in a high-fat diet (HFD) induced diabetic osteoporosis. HFD-induced type 2 diabetes mellitus which is characterized by hyperglycemia and insulin resistance blocks the AMPK pathway, inhibits BMP-2 production, Smad phosphorylation, and bone forming RunX2 gene expression. This results in the alteration of downstream biomarkers including RANKL/OPG, ALP, and OCN which subsequently inhibits bone formation. Another pathway is the hyperglycemia upregulated DPP-4 enzyme which reduces incretins thereby increasing the production of bone marrow adipose tissue which further upregulates pro-inflammatory cytokines and sclerostin, which subsequently inactivates the Wnt signaling pathway. Inactivated Wnt pathway and adipocyte-induced PPARϒ overexpression increase bone resorption by altering bone turnover markers like RANKL/OPG, ALP, TRAP, and OCN. HFD-induced high leptin levels also cause adipocyte formation and reduced bone formation. Treatment with linagliptin (DPP-4 inhibitor) and its combination with metformin mitigate hyperglycemia and leptin production while increasing incretins and bone formation. This is possibly achieved *via* activating AMPK and Wnt pathways as confirmed by increased BMP-2 expression and reduced sclerostin respectively. L, Linagliptin; M, Metformin; DPP-4, Dipeptidyl peptidase-4; Wnt, Wingless and Int. protein; AMPK, AMP-activated protein kinase; BMP-2, Bone morphogenetic protein; RunX-2, Runt related transcription factor 2; RANKL/OPG, Receptor activator of nuclear factor kappa-B ligand/Osteoprotegerin; ALP, Alkaline phosphatase; TRAP, Tartrate Resistant Acid Phosphatase; OCN, Osteocalcin; PPARϒ, Peroxisome proliferators–activated receptor γ; GLP, Glucagon-like peptide; GIP, Gastric inhibitory polypeptide; TNF-α, Tumor Necrosis Factor-alpha, IL-6, Interleukins-6, IL-β, Interleukins.

Thus, based on our observations, it may be inferred that increased BMP-2 levels and reduced sclerostin levels result in alteration of other bone turnover biomarkers such as bone ALP, osteocalcin, TRAP, and serum calcium which might be responsible for the bone-protective effects of linagliptin and its combination with metformin. A schematic representation of the possible mechanistic explanation is portrayed in **Figure 11**.

High fat-linked obesity and higher adipose tissue accelerate the production of inflammatory cytokines, which results in insulin resistance, diabetes, and osteoclastogenesis (86, 87). We observed that pro-inflammatory cytokines such as TNF-α, IL-6, and IL-1β were increased following HFD-induced diabetes (71, 88). Linagliptin, metformin, and their combination reduced TNF-α and IL-1β but not IL-6 levels. This is in line with other *in-vitro* and *in-vivo* studies which confirm that anti-diabetic drugs metformin and linagliptin inhibit pro-inflammatory cytokines (89–92).

Overconsumption of a diet rich in fats is known to accelerate adipocyte overproduction which subsequently boosts leptin levels, resulting in high bone resorption (93–95). Likewise, excessive cholesterol and triglyceride are also corroborated to increase the risk of osteoporosis in patients (96). Present study findings that HFD raises leptin, cholesterol, and triglyceride levels are in agreement with the other preclinical studies (70,71). The combination of linagliptin and metformin significantly depleted serum leptin, cholesterol, and triglycerides agree with the past studies which unveil the leptin and lipid-lowering effect of metformin and DPP-4 inhibitors (97–99).

## Conclusion

In summary, we conclude that improvement in bone architecture, BMD, bone turnover biomarkers, and bone histopathology by the combination of linagliptin and metformin shows its bone protective potential in reversing HFD-induced diabetic osteoporosis which is possibly mediated through modulation of BMP-2 and sclerostin. The effect was better in the combination as compared to the individual drugs alone. Our findings need to be explored further for the amelioration of diabetes-associated bone deficits clinically where the combination could be preferred in those diabetic patients with a risk factor for osteoporosis. However, future studies should continue to explore and confirm the exact mechanism involved in the osteogenic effects of the combination of linagliptin and metformin by analyzing the expression of several genes involved in the BMP-2 and sclerostin regulated AMPK and Wnt/β catenin signaling pathways.

## Data Availability Statement

The raw data supporting the conclusions of this article will be made available by the authors, without undue reservation.

## Ethics Statement

The animal study was reviewed and approved by Jamia Hamdard Animal Ethics Committee affiliated from Jamia Hamdard.

## Author Contributions

Both authors equally contributed to designing the experiment. NN performed all experiments, collected data, performed the statistical analysis, and wrote the manuscript. DV has supervised the research work and edited the manuscript for the intellectual and scientific content. All authors contributed to the final shaping of the article and approved the submitted version.

## Funding

This work was supported by the Indian Council of Medical Research (ICMR), under grant approval No: 45/33/2019-NANO-BMS. The authors are thankful to UGC SAP DRS-2 grant for the micro-CT facility in Neurobehavioral Pharmacology Laboratory.

## Conflict of Interest

The authors declare that the research was conducted in the absence of any commercial or financial relationships that could be construed as a potential conflict of interest.

## Publisher’s Note

All claims expressed in this article are solely those of the authors and do not necessarily represent those of their affiliated organizations, or those of the publisher, the editors and the reviewers. Any product that may be evaluated in this article, or claim that may be made by its manufacturer, is not guaranteed or endorsed by the publisher.
